# Overcoming barriers: a review on innovations in drug delivery to the middle and inner ear

**DOI:** 10.3389/fphar.2023.1207141

**Published:** 2023-10-19

**Authors:** Derek S. Delaney, Lawrence J. Liew, Joey Lye, Marcus D. Atlas, Elaine Y. M. Wong

**Affiliations:** ^1^ Hearing Therapeutics, Ear Science Institute Australia, Nedlands, WA, Australia; ^2^ Faculty of Health Sciences, Curtin Health Innovation Research Institute, Curtin University, Bentley, WA, Australia; ^3^ Centre for Ear Sciences, Medical School, The University of Western Australia, Nedlands, WA, Australia; ^4^ Faculty of Health Sciences, Curtin Medical School, Curtin University, Bentley, WA, Australia

**Keywords:** drug delivery, inner ear, middle ear, hydrogel, nanoparticle, biomaterials, microdevices

## Abstract

Despite significant advances in the development of therapeutics for hearing loss, drug delivery to the middle and inner ear remains a challenge. As conventional oral or intravascular administration are ineffective due to poor bioavailability and impermeability of the blood-labyrinth-barrier, localized delivery is becoming a preferable approach for certain drugs. Even then, localized delivery to the ear precludes continual drug delivery due to the invasive and potentially traumatic procedures required to access the middle and inner ear. To address this, the preclinical development of controlled release therapeutics and drug delivery devices have greatly advanced, with some now showing promise clinically. This review will discuss the existing challenges in drug development for treating the most prevalent and damaging hearing disorders, in particular otitis media, perforation of the tympanic membrane, cholesteatoma and sensorineural hearing loss. We will then address novel developments in drug delivery that address these including novel controlled release therapeutics such as hydrogel and nanotechnology and finally, novel device delivery approaches such as microfluidic systems and cochlear prosthesis-mediated delivery. The aim of this review is to investigate how drugs can reach the middle and inner ear more efficiently and how recent innovations could be applied in aiding drug delivery in certain pathologic contexts.

## 1 Introduction

**FIGURE 1:**
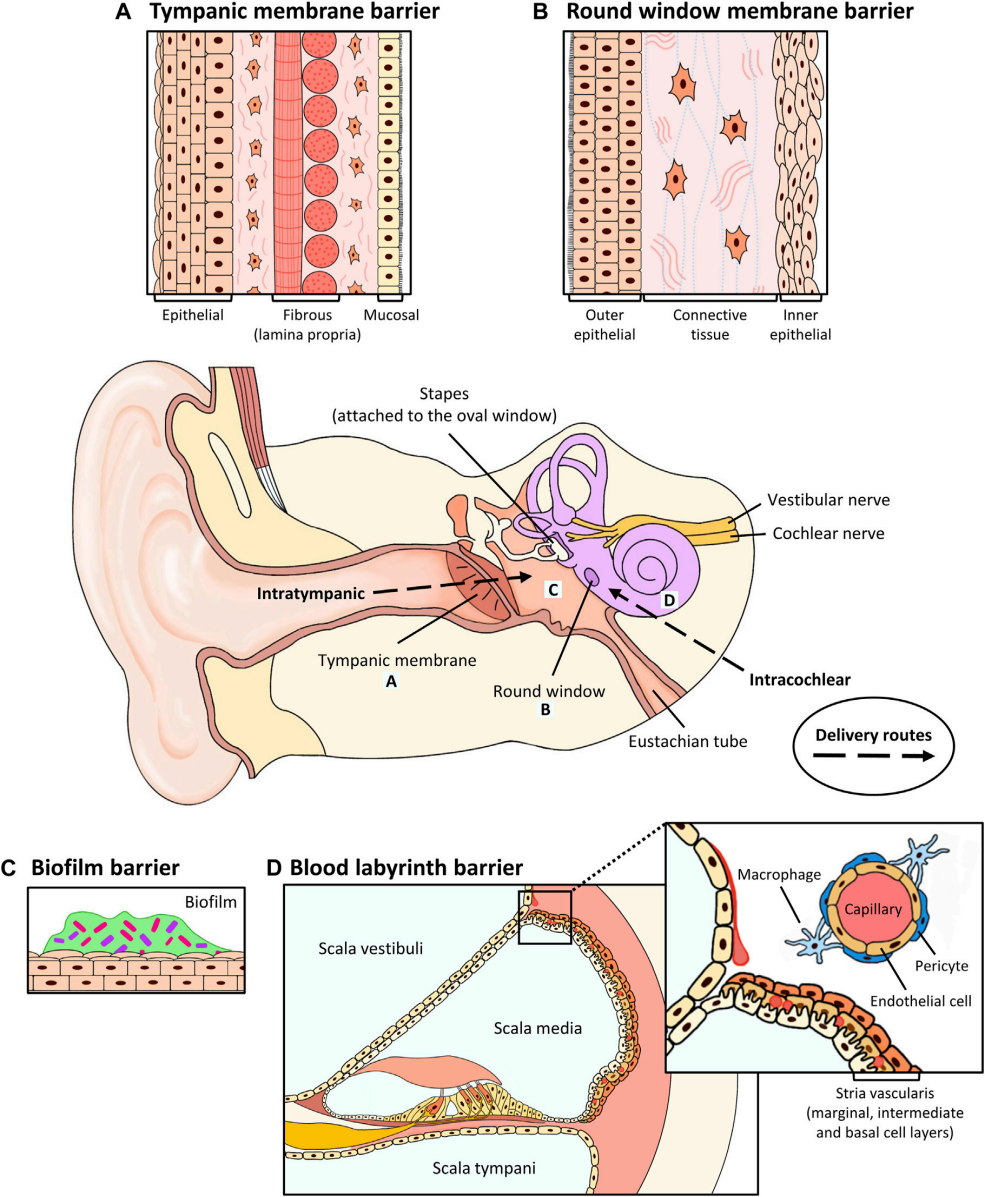
Anatomical barriers to drug penetration in the middle and inner ear, and routes of local delivery. **(A)** Tympanic membrane. The TM consists of a stratified outer epithelium, a lamina propria (radial and circular fibrous) containing fibroblasts and a thin layer of mucosal epithelium. Intratympanic and topical drugs are administered here. **(B)** Round window membrane. The RWM consists of an outer epithelium, a connective tissue layer containing fibroblasts, collagen and elastic fibers and an inner epithelium. Intratympanic drugs are administered here. **(C)** Biofilm. Biofilm forms an encapsulated layer of extracellular proteins to protect pathogenic microorganisms against antibiotics. **(D)** Blood labyrinth barrier. The stria vascularis contains 3 cell layers: the marginal, intermediate and basal cell layers. The BLB consists of pericytes, endothelial cells and macrophages and surrounds the capillaries that are embedded within the intermediate cell layers of the stria vascularis. As shown in the inset in **(D)**, an enlarged view is shown to illustrate the cellular component of the BLB system. Systemically administered drugs would need to cross the BLB to access the inner ear.

Hearing loss is one of the most common sensory disabilities, with the World Health Organization estimating over 1.5 billion people worldwide to currently experience some form of hearing impairment (Chadha et al., 2021). The ear is a highly compartmentalized organ with anatomical barriers that preclude conventional drug delivery both locally to affected sites or systemically due to the blood labyrinth barrier (BLB; Figure 1). Systemically administered drugs additionally risk being poorly bioavailable or having unwanted effects in other organs. Local administration is preferred to solve both issues, however the procedures involved are significantly more invasive. This presents a clinical challenge for the administration of multiple doses and some newer technologies such as gene therapy, which shows great promise in the treatment of hereditary conditions and the regeneration of hair cells (Nyberg et al., 2019; Szeto et al., 2019; French et al., 2020). We will briefly discuss the etiology of otitis media, perforation of the tympanic membrane and cholesteatoma of the middle ear and sensorineural hearing loss of the cochlea; then discuss current and emerging treatments and recent progress in improving drug delivery to the middle and inner ear for these diseases.

## 2 Diseases of the middle ear

Otitis media (OM), perforation of the tympanic membrane (TM), and cholesteatoma are the most commonly reported middle ear diseases in clinical practice. Common signs and symptoms such as pain, fever, sudden hearing loss, and the sensation of fullness in the ear may help identify middle ear diseases, but additional approaches such as otoscopy and magnetic resonance imaging (MRI) are often required to confirm the diagnosis (Trojanowska et al., 2012; Lo and Nemec, 2015).

### 2.1 Otitis media

OM represents a spectrum of middle ear inflammatory diseases which can be classified into acute otitis media (AOM), otitis media with effusion (OME, also known as glue ear), and chronic suppurative otitis media (CSOM), which is one of the most severe middle ear diseases. The common symptoms for AOM and OME include earache, hearing loss, swelling and bulging of the TM, and the accumulation of fluid behind the TM. Meanwhile, CSOM is characterized by chronic inflammation and recurrent purulent discharge through a perforated TM (Leach et al., 2021).

OM is one of the leading causes of permanent hearing loss in children and a significant disease burden in developing countries and several OM-prone populations such as Australian Aboriginals (Li et al., 2015; DeAntonio et al., 2016; DeLacy et al., 2020). Children and adolescents are particularly susceptible to OM, and two of the most accepted theories are an immature Eustachian tube (structural and functional) that is prone to blockage; and underdeveloped innate and adaptive immune systems (Bluestone, 2008; Pichichero, 2020).

Bacterial pathogens such as *Streptococcus pneumoniae*, *Haemophilus influenzae*, *Moraxella catarrhalis* and *Pseudomonas aeruginosa* are the most common cause of OM, but it can also be caused by *Influenza* viruses or fungal species such as *Aspergillus* (Mittal et al., 2015; Bhutta et al., 2017; Rowe et al., 2019). There is increasing evidence that biofilm, a microhabitat of microorganisms entrapped in self-produced extracellular proteins, contributes to the pathogenesis of OM. Biofilms are difficult to eradicate as they are resistant to antibiotics and firmly adhered to the middle ear epithelium, ossicles and tympanostomy tube (Mittal et al., 2015; Mittal et al., 2018; Silva and Sillankorva, 2019).

### 2.2 Tympanic membrane perforations

Perforations can occur due to OM or trauma to the TM, such as the piercing of the TM by a sharp object or a sudden change in pressure (barotrauma) (Spandow et al., 1996; Wang et al., 2014a). Most traumatic or acute TM perforations heal spontaneously within 1–2 weeks, but those that fail to heal within 3 months are considered chronic TM perforations which require surgical intervention (Tan et al., 2016). The mechanism that underlies the development of chronic TM perforation is unknown, but studies have identified risk factors that are linked to delayed healing of TM perforations, including perforation size and location (Lou et al., 2011; Tan et al., 2016), Eustachian tube dysfunction (Varsak and Santa Maria, 2016; Paltura et al., 2017) and long-term tympanostomy tube usage (Kay et al., 2001; Alrwisan et al., 2016; Knutsson et al., 2018).

### 2.3 Cholesteatoma

Cholesteatoma is described as an inflammatory, non-malignant (and pearl-like) epithelial lesion in the middle ear cavity that can lead to conductive hearing loss, erosion of the ossicles, and other intracranial complications. Congenital cholesteatoma is rare and contributes to 1%–5% of all cholesteatoma cases; and is often detected within the first decade of life (Mansour et al., 2018; Jenks et al., 2022). The development of congenital cholesteatoma is thought to be due to the entrapment of epithelial remnants during embryogenesis, but the actual mechanism that drives this process remains under debate (Gilberto et al., 2020). Meanwhile, the more common acquired cholesteatoma is categorized into either primary acquired, which is caused by a retraction pocket in the TM; or secondary acquired, which is thought to be caused by an abnormal growth of the TM epithelial layer in the middle ear through a perforation (Cho et al., 2016; Clark et al., 2016). The pathogenesis of acquired cholesteatoma can be grouped into four theories: retraction pocket or invagination theory; epithelial invasion theory; squamous metaplasia theory and basal cell hyperplasia theory (Kuo et al., 2015; Hamed et al., 2016). More recently, the dysregulation of cell signaling and immunological pathways such as EGF; IL-6/JAK/STAT3, MicroRNA-21, Notch and TNF signaling have been identified as additional potential contributors (Hilton et al., 2011; Liu et al., 2014; Chen et al., 2016; Xie et al., 2016; Fukuda et al., 2021).

## 3 Sensorineural hearing loss

The most common type of hearing impairment is sensorineural hearing loss (SNHL). Patients with SNHL perceive sound as muted and distorted when compared to other forms of hearing loss, and it is caused by the degeneration or malfunction of cochlear hair cells or spiral ganglion nerves of the inner ear. This can be caused by a wide variety of environmental insults such as excessive noise, which is the second largest cause of SNHL, the administration of ototoxic drugs, exposure to ototoxic chemicals including certain solvents and metals, intrauterine infections including toxoplasmosis, cytomegalovirus and human immunodeficiency virus, birth asphyxia and hyperbilirubinemia. Genetics also play a role in more sporadic, yet highly severe forms of SNHL where aspects of cochlear development or structure are crippled by mutations to certain genes. These mutations can be inherited or, less commonly, arise *de novo* in the developing cochlea. In the following subsections, we summarize the etiology of the more characterized common or severe forms of SNHL: hereditary, noise-induced, age-related, drug-induced and idiopathic.

### 3.1 Hereditary sensorineural hearing loss

Hereditary SNHL is caused by the inheritance of one or more gene mutations from biological parents that carry defective alleles, and affects about 1 per 1,000 newborns with profound deafness (Gettelfinger and Dahl, 2018). Mutations cause the translation of malfunctional proteins that can no longer fulfil their native function and cohesively work with other components of the auditory system. Most hereditary-related deafness is monogenic, with approximately 80% of cases being autosomal recessive, although other modes of transmission such as autosomal dominant and X-linked can also occur (Schrujver, 2004). There have been 124 deafness-associated genes identified to date and some of the most prevalent include MYO7A and USH2A which are associated with Usher syndrome, PAX3 and SOX10 which are associated with Waardenburg syndrome, and PDS which is associated with Pendred syndrome (Nishio et al., 2015; Van Camp and Smith, 2023).

### 3.2 Noise-induced hearing loss

Noise-induced hearing loss (NIHL) is caused by progressive over-exposure to noise. NIHL affects all ages, however there is an increasing prevalence of NIHL occurring in younger populations from recreational noise exposure, for example from listening to music using headphones for prolonged periods, with over 50% of those aged 12–35 being estimated to risk developing NIHL (Chadha et al., 2021). Occupational NIHL from jobs involving prolonged exposure to loud noise, including from power tools, automotive, gunfire and music, has been attributed to 16% of worldwide severe hearing loss, with men shown to be more susceptible than women (Nelson et al., 2005). Long-term noise exposure can risk damage to the auditory structures which deteriorates cochlear hair cells and spiral ganglion nerves. Moreover, certain genetic variants have been identified clinically and experimentally to increase susceptibility to NIHL (Beaulac et al., 2021; Jiang et al., 2021). The early signs of NIHL are the loss of high frequency hearing and decreased ability to distinguish speech over background noises, and is often accompanied by tinnitus (Le et al., 2017).

### 3.3 Age-related hearing loss

Age-related hearing loss (ARHL), or presbycusis, affects the elderly as they gradually lose hearing in both ears and over 65% of adults over 60 years of age have been shown to be affected (Cunningham and Tucci, 2017; Chadha et al., 2021). ARHL usually has a complex etiology which encompasses lifestyle aspects such as noise, but does have some other defined risk factors such as smoking, ototoxic drug use and hypertension (Gates and Mills, 2005; Bowl and Dawson, 2019). Moreover, ARHL has been shown to cluster in families with prevalent cases (Gates et al., 1999). In terms of pathological features, four classifications of ARHL have been proposed from post-mortem human tissue examination, including ARHL from progressive hair cell loss, sensory and neural degeneration, loss of stria vascularis integrity and stiffening of the basilar membrane (Schuknecht, 1964; Schuknecht and Gacek, 1993). Additional etiologies have been proposed based on post-mortem human tissue analyses and animal models, involving oxidative stress, chronic inflammation, and loss of lateral wall fibrocytes (Ohlemiller and Gagnon, 2004; Suzuki et al., 2006; Tawfik et al., 2020), but it is likely that the clinical presentation will involve more than one of these causes.

### 3.4 Drug-induced hearing loss

Drug-induced hearing loss occurs when patients take drugs that have ototoxic side effects. Aminoglycoside and macrolide antibiotics in particular, such as gentamicin and azithromycin, are commonly used in the treatment of Meniere’s disease and in developing countries for other diseases, however they are highly ototoxic (Rauch et al., 2011; Rybak et al., 2021). Aminoglycosides can permeate through the BLB or round/oval window (when administered intratympanically for Meniere’s disease) due to their small molecular size, and accumulate in the apical side of hair cells through mechanoelectrical transduction (MET) channels or by endocytosis (Nyberg et al., 2019). When MET channels are stimulated by sound, antibiotics can enter them and exert cytotoxicity on hair cells through a yet unclear mechanism (Alharazneh et al., 2011; Zimmerman and Lahav, 2013). For ototoxic chemotherapies, cisplatin is perhaps the most widely prescribed example (Greene et al., 2015; Frisina et al., 2016). Cisplatin increases the generation of reactive oxygen species by inhibiting peroxidase enzymes which progressively damages hair cells over time, leading to SNHL (Sheth et al., 2017). Other ototoxic drugs include the antimalarial drug quinine and loop diuretics such as furosemide and acetylsalicylic acid (Tange et al., 1997; Ding et al., 2016). Furthermore, ototoxicity can be exacerbated by interactions with certain drugs, genetic polymorphisms and cochlear inflammation (Barbieri et al., 2019; Coffin et al., 2021).

### 3.5 Idiopathic sudden sensorineural hearing loss

Sudden sensorineural hearing loss (SSNHL) is characterized by the presentation of patients with at least 30 dB hearing loss occurring within 72 h. The incidence in the United States was reported to be 5 to 20 per 100,000 people with the vast majority of cases being unilateral and having an unknown etiology, being termed idiopathic (Kuhn et al., 2011). Bilateral SSNHL is less prevalent, comprising an estimated 4.9% of SSNHL cases, however is not usually idiopathic and is secondary to other complications including cancer, vascular disorders and autoimmune disease (Sara et al., 2013). Idiopathic SSNHL does spontaneously resolve in 45%–65% of patients, however controversy remains on potential etiologies and best course of treatment (Conlin and Parnes, 2007; Kuhn et al., 2011).

## 4 Current and emerging therapeutic options

### 4.1 Current interventions for middle ear diseases

Middle ear diseases such AOM and OME are most commonly managed with supportive care such as painkillers or oral antibiotics such as Amoxicillin, but vaccination against common middle ear pathogens such as *S. pneunomiae* and *H*. *influenzae* has become standard practice in some countries (Martinovich et al., 2021; Clark et al., 2022; Izurieta et al., 2022). The treatment of CSOM remains a clinical challenge and often requires the combination of oral antibiotics, eardrops, and surgery due to the presence of antibiotic-resistant bacteria and bacterial biofilm (Mittal et al., 2018; Leach et al., 2021). Moreover, recurrence of the infection can occur from the presence of persister cells, a phenotype of bacteria within biofilms that exhibit low metabolic activity (Tolker-Nielsen, 2014; Fisher et al., 2017). Persister cells have been shown to escape the initial course of antibiotics and resume growth and metabolism once the administration is halted (Khomtchouk et al., 2020; Santa Maria et al., 2021).

Acute or traumatic TM perforations are most often treated with supportive care as with OM, to minimize pain and prevent infection (Orji and Agu, 2008; Lieberthal et al., 2013). Surgery is only indicated for chronic TM perforations (over 3 months) and this procedure is called myringoplasty (or tympanoplasty if ossicles are involved), which involves the placement of an autologous graft (typically temporalis fascia or auricular cartilage) over or under the defect to encourage wound healing and restore hearing. The success rate for such surgery is approximately 87% (Tan et al., 2016), but the harvesting of autologous grafts is associated with donor site morbidity (infection and pain) and higher operation cost (Villar-Fernandez and Lopez-Escamez, 2015; Sainsbury et al., 2022).

For cholesteatoma, surgical excision remains the only curative approach and this is often performed in conjunction with reconstruction surgeries (e.g., mastoid wall reconstruction) to repair damage caused by the lesion and surgical procedure (Mansour et al., 2018). Unfortunately, the recurrence rate for cholesteatoma is high with 10%–40% of patients requiring repeated surgery (Britze et al., 2017; Angeli et al., 2020; Bächinger et al., 2021). The recurrence of cholesteatoma may be affected by the type of surgical procedure and is thought to vary between congenital and acquired disease (Edfeldt et al., 2012; Morita et al., 2017; van der Toom et al., 2021; Adriaansens et al., 2022).

### 4.2 Emerging interventions for middle ear diseases

Bacteriophage or viral-based treatment has emerged as an alternative strategy to counteract the increasing threat of antibiotic resistance (Gordillo Altamirano and Barr, 2019). Wright et al. (2009) conducted the first clinical trial on the safety of a bacteriophage preparation against *P. aeruginosa* in patients with chronic OM. Overall, the treatment did not cause any severe side effects, significantly lowered the bacteria count (CFU per gram) and improved the clinical scores of patients. In a later clinical trial, bacteriophage therapy was conducted in burns patients infected with *P. aeruginosa* (Jault et al., 2019). Unlike the previous trial, a daily topical application of a cocktail of 12 bacteriophages suspended in saline solution was compared to the standard of care (1% sulfadiazine silver emulsion cream) over 7 days. Overall, the treatment was well tolerated, but there was no significant clinical improvement or reduction in bacterial load when compared to standard of care. This study also highlighted a few challenges associated with bacteriophage treatment, including the stability and titer of bacteriophage after manufacturing and concerns about bacterial resistance to bacteriophages. Overall, bacteriophage-based treatment remains an exciting and promising therapeutic approach for the treatment of OM and warrants further investigation.

There has been an increased interest in the development of effective anti-biofilm technology as a preventative and treatment approach. One of the most promising treatment targets is Type IV pili (Tfp), a type of proteinous appendage on the surface of most bacterial species, which plays a critical role in mechanosensing, motility, and the formation of biofilm (Lee et al., 2018; Horna et al., 2019). Novotny and colleagues have successfully developed a transcutaneous immunization strategy against Tfp and showed both *in vitro* and in animal studies the efficacy of anti-Tfp antibodies in destabilization of *H. Influenzae* biofilm and the eradication of the pathogen (Novotny et al., 2013; Novotny et al., 2015; Novotny et al., 2016; Novotny et al., 2021). Anti-Tfp treatment has also been shown to be effective against other middle ear pathogens such as *M*. *catarrhalis* (Mokrzan et al., 2018). More importantly, the antibody-mediated release bacteria (from the biofilm) were found to be more susceptible to antibiotic killing as compared to their planktonic (free-living) counterparts (Mokrzan et al., 2018; Mokrzan et al., 2020). Other promising strategies to tackle problems associated with biofilm include antibody treatment against bacterial DNABII protein, a key structural component of the biofilm (Barron et al., 2020; Novotny et al., 2021). Dornase alfa, a recombinant human deoxyribonuclease that breaks down DNA, has also been shown to reduce the development of otorrhea in pediatric patients undergoing tympanostomy (Thornton et al., 2013; Chan et al., 2018).

For the repair of TM, much research has been focused on developing biocompatible scaffolds using natural or synthetic materials such as collagen, chitosan, decellularized tissue and polylactic acid (PLA) (Hussain and Pei, 2021; Sainsbury et al., 2022). Some of the current commercially available scaffolding materials indicated for myringoplasty are EpiFilm^®^ (Medtronic, Ireland), which is a hyaluronic acid-derived implantable device, and acellular dermal allografts such as Alloderm (Allergan, Ireland) (Vos et al., 2005; Sayin et al., 2013).

Otherwise, adjuvant therapies using topical application of biomolecules are becoming popular in addition to myringoplasty to enhance the healing of perforations. The most commonly used biomolecules are growth factors such as epidermal growth factor (EGF), basic fibroblast growth factor (bFGF) (Hong et al., 2013; Sainsbury et al., 2022). The efficacy of EGF on the closure of TM perforations has been investigated predominantly in acute/traumatic perforations. (Lou et al., 2016; Lou and Lou, 2017; Lou and Lou, 2018b). Despite the positive outcomes, the benefits of EGF on the repair of chronic TM perforation remain unclear due to the lack of well-controlled clinical studies.

Multiple clinical trials have been conducted to study the effects of bFGF on both acute and chronic TM perforations. For acute TM perforations, topical drops administration is the most commonly used approach and is associated with improved healing outcomes when compared to untreated controls (Huang et al., 2020). For chronic perforations, the most widely used approach for bFGF therapy was developed by Kanemaru and colleagues in which a bFGF-impregnated scaffold (Gelfoam) is applied over the perforation, which is then sealed with fibrin glue (Kanemaru et al., 2011). Clinical trials conducted in Japan have reported closure rates of 62%–100% in chronic perforations treated with bFGF (Hakuba et al., 2003; Hakuba et al., 2010; Hakuba et al., 2013; Hakuba et al., 2015). In contrast, a recent Phase 2 clinical trial comparing chronic TM perforations treated with bFGF or placebo (water) reported no statistical difference in closure rates between the two treatment groups (Santos et al., 2020). Besides that, there have been concerns about the safety of bFGF as it was found to lead to other middle ear disorders such as myringitis and cholesteatoma (Hakuba et al., 2013; Lou and Lou, 2018a).

Platelet-rich-plasma (PRP), a type of blood product that is rich in platelets and bioactive molecules, has been shown to be a promising alternative treatment option for tissue repair and regeneration (Amable et al., 2013; Everts et al., 2020; Huang et al., 2021). PRP has also emerged as a promising treatment for chronic TM perforations with 8 clinical trials conducted in the past 10 years (El-Anwar et al., 2015; Fawzy et al., 2018; Fouad et al., 2018; Yadav et al., 2018; Mandour et al., 2019; Anwar et al., 2020; Ersözlü and Gultekin, 2020; Taneja, 2020). The closure rate ranged from 85.7% to 100% in the PRP treatment group while a 55%–92% closure rate was reported in the control group. It is noteworthy that PRP was used in conjunction with a scaffolding material including a fat graft, temporalis fascia, or conchal perichondrium. Therefore, the real therapeutic benefit of PRP as a stand-alone treatment remains unclear.

Overall, adjuvant therapy using biomolecules with or without myringoplasty remains an attractive avenue for the management of TM perforations with research expanded beyond the traditional growth factors (Shen et al., 2014; Araujo et al., 2016). More recently, a Phase 1 clinical trial investigating the effect of a recombinant heparin-binding epidermal growth factor-like growth factor (HB-EGF) (dubbed ASP0598 Otic Solution) on the healing of chronic TM perforations has started patient recruitment in multiple locations in the United States (NCT04305184, clinicaltrials.gov).

### 4.3 Current interventions for sensorineural hearing loss

Auditory rehabilitation with hearing devices or cochlear implants remains the primary treatment approach for SNHL. These technologies can significantly aid in communication, yet they are still unable to mimic the quality of natural hearing and, more importantly, they do not treat the underlying cause of the hearing loss (Beck and Le Goff, 2018; Solheim et al., 2018).

Hearing aid technology has advanced greatly over the past decade, with innovations allowing customization of every device to fit the unique hearing needs of each patient. These devices are designed to amplify sounds and restore hearing, however no currently available devices can provide sound quality comparable to that of a healthy cochlea. Some hearing aids are capable of selectively reducing background noise while maintaining access to all distinct speech sounds, but this typically requires the user to select the voice they wish to focus on and are usually ineffective in group settings. Moreover, hearing aids still rely on functional hair cells for sound transduction (Geleoc and Holt, 2014).

In contrast, cochlear implants have proven to be a successful therapeutic approach for patients, even for those with abnormal hair cells and severe hearing impairment. There is clear evidence showing rapid development in oral communication and auditory skills in infants and children with SNHL with cochlear implants (Mok et al., 2010; Dettman et al., 2016; Hoff et al., 2019; Dettman et al., 2021). Moreover, adult patients with cochlear implants were shown to have improved speech outcomes and quality of life (Santa Maria et al., 2013; Völter et al., 2020). However, evidence in patients and animal models show the surgical installation procedure risks chronic cochlear inflammation or fibrosis, which reduces the effectiveness of the implant over time (Wilk et al., 2016; Foggia et al., 2019).

### 4.4 Emerging interventions for sensorineural hearing loss

There is currently a wide spectrum of drug types undergoing clinical trials for different types of SNHL, which has been recently comprehensively summarized by Isherwood et al. (2022). In recent years, strategies aiming to manipulate hair cell differentiation pathways have garnered significant interest. For example, hair cell differentiation is shown to be accompanied by *ATOH1* gene expression, crucial for progenitor cells residing in the organ of Corti to be directed to differentiate into a non-sensory or sensory lineage (Cai et al., 2013; Driver et al., 2013). Currently, the CGF166 adenoviral vector contains *ATOH1* cDNA transcript to replace absent hair cells has completed Phase 2 investigation (NCT02132130, clinicaltrials.gov). However, most recently reported novel otoprotective, regenerative and gene replacement treatments remain too early in their stage of development to be applied clinically.

For cisplatin-induced hearing loss, sodium thiosulfate has recently been approved by the US Food and Drug Administration as an otoprotective drug. As mentioned, cisplatin is a highly ototoxic drug, with children and adolescents undergoing cisplatin treatment being particularly vulnerable to cisplatin-induced hearing loss (Orgel et al., 2022). Sodium thiosulfate has been demonstrated in animal models and clinical trials to abrogate cisplatin-induced ototoxicity, thought to occur by directly chelating cisplatin (Videhult et al., 2006; Brock et al., 2018). However, as sodium thiosulfate directly binds cisplatin and is currently intravenously injected, this may risk decreasing the efficacy of the chemotherapy on the cancer itself. Methods of locally administering sodium thiosulfate to the inner ear are currently being investigated to reduce this risk and will be discussed in later sections.

Another developing approach to treating inner ear disorders is the application of exosomes. Exosomes are a subset of extracellular vesicle derived from the budding of endosomes from the plasma membrane, incorporating transmembrane proteins and lipids, and range from ∼30 to 160 nm in diameter. The precise physiological role of exosomes is unclear, however they have been demonstrated to have pleiotropic roles in cell-cell communication, signaling, proliferation, angiogenesis, immune response and metabolism and have been shown to carry proteins, nucleic acids and metabolites (Kalluri and LeBleu, 2020; Warnecke et al., 2022). Exosomes have been recently identified, isolated and characterized from the perilymph of adult patients with various forms of SNHL or mixed hearing loss, with the study suggesting a hair cell origin of the exosomes by their expression of MYO7A (Zhuang et al., 2021). On the therapeutic side, a study from Athanasia Warnecke and colleagues demonstrated protective effects of bone marrow mesenchymal stromal cell-derived extracellular vesicles on a mouse model of NIHL, which additionally promoted neurite outgrowth *in vitro* (Warnecke et al., 2020). This was followed up by a study demonstrating the safety of implanted extracellular vesicles in a Meniere’s disease patient that had undergone cochlear implantation 24 months post-operation (Warnecke et al., 2021). Exosomes thus represent an interesting potential avenue of biological, yet cell-free treatment of inner ear disorders.

## 5 Challenges in drug delivery to the middle and inner ear

Oral or intravenous delivery of antibiotics are the standard approach for treating middle ear disorders, but this has been shown to risk poor bioavailability and side effects such as diarrhea (Chee et al., 2016). Topical treatments such as ciprofloxacin/dexamethasone ear drops or Otiprio^®^ are efficacious for OM, however they are indicated exclusively for OM with perforated TM (e.g., post tympanostomy or CSOM) (van Dongen et al., 2014). While animal and human cadaveric studies have showed that intact TMs are readily permeable to ciprofloxacin (Yang et al., 2016; Yang et al., 2018b; Early et al., 2021), it remains unclear whether such results can be achieved in a clinical setting. Apart from this physical barrier, bacterial biofilm formed during OM creates a second physical-chemical barrier that hinders treatment (Silva and Sillankorva, 2019).

Similarly, the cochlea is among the most difficult organs to deliver drugs to by conventional systemic routes. It is encased by the petrous bone, which is the densest bone in the human body, and is only accessible to the circulation via the BLB. As with the treatment of OM, drugs administered systemically generally accumulate poorly in the inner ear and can exert unwanted effects elsewhere due to the aforementioned barriers (Rauch et al., 2011). Therefore, to effectively deliver drugs to the cochlea, more invasive localized delivery procedures are required to ensure bioavailability and avoid unwanted systemic effects. These are injections by intratympanic and intracochlear routes (Figure 1). While being more invasive and potentially traumatic, these injections allow more direct administration of drug to the inner ear. Intratympanic injection generally relies on the permeation of the therapeutic agent across the round window (RWM) or oval window membrane, which are more permeable than the BLB or TM. However, it still presents a delivery challenge as most drugs remain poorly bioavailable in the inner ear due to clearance across the BLB or Eustachian canal (Salt and Plontke, 2018). Non-specific binding or aggregation can also occur due to the positively-charged perilymph if the molecule is negatively charged, for example DNA or RNA. A recent study comparing intratympanic and intracochlear delivery of a novel supraparticle encapsulated neurotrophin-3 has showed the latter to be a superior approach, but may not be practical for drugs that require multiple doses due to the risk of scarring or inner ear fibrosis (Gunewardene et al., 2022). Another alternative injection method that has become more prevalent is postauricular or retroauricular injection which delivers drug to the postauricular region of the ear without perforating the tympanic membrane. This has the advantage of avoiding potential side effects of intratympanic injection such as persistent pain, otitis media or acute or chronic tympanic perforation. (Li et al., 2021; Chen et al., 2022c; Xie et al., 2023). However, it is currently not performed worldwide and it remains unclear how drugs reach the inner ear from the postauricular region (Qiu et al., 2022).

To counteract these physiological challenges, there has recently been considerable progress in the use of biomaterials such as hydrogels and nanotechnology. Formulations involving these biomaterials are aimed at enhancing drug bioavailability and providing controlled and sustained release. Sustained release can be triggered either upon temperature change, phase change, implantation, pH changes or administration of a secondary agent (You and Auguste, 2009; Lajud et al., 2015; Li and Mooney, 2016; Dai et al., 2018). The following sections will address how hydrogels and nanotechnology are being employed to aid drug delivery and facilitate the delivery of novel and repurposed therapeutics into the middle and inner ear. We additionally have summarized examples of biomaterial-based drug delivery platforms in Table 1.

**TABLE 1:** Examples of proposed biomaterial-based therapies for sensorineural hearing loss undergoing preclinical studies.

Biomaterial type	Formulation	Administration route(s) tested	Example of proposed intervention	Model	References
Hydrogel	Chitosan	Intratympanic RWM application	CGP/chitosan hydrogel used to deliver PEG nanoparticles containing JNK inhibitor functionalized with prestin-targeting peptide for outer hair cell targeting	Mouse	Kayyali et al. (2018)
	GelMA	Intratympanic RWM application	Hydrogel microspheres containing ebselen liposomes, functionalized with polydopamine for cell adhesion	Mouse	Chen et al. (2022d)
	Hyaluronic acid	Intratympanic RWM application	Hyaluronic acid hydrogel containing brimapitide, an inhibitor of JNK	Chinchillla	(Eshraghi et al., 2018)
	PEG	Silicone tubes linking middle to inner ear	NCOsP(EO-stat-PO) hydrogel containing dexamethasone 21-phosphate disodium salt	Guinea pig	Hütten et al. (2014)
	PLGA	Intratympanic RWM application	PLGA-PEG-PLGA hydrogel containing cidofovir	Guinea pig	Feng et al. (2014)
	Poloxamer	Intratympanic RWM application	Poloxamer 407/cyclodextrin hydrogel containing apoptasome inhibitor LPT99	Rat	Murillo-Cuesta et al. (2021)
Lipid	Liposomes	Intratympanic	Liposome nanocarriers containing gadolinium for MRI contrast imaging	Rat	Zou et al. (2017)
	Solid-lipid	Intratympanic, systemic	Solid-lipid nanoparticles containing clozapine or edavarone	Guinea pig	Gao et al. (2015), Wang et al. (2020)
Polymeric	Lipid polymer	RWM application	PEG/DMPC phospholipid nanoparticle containing astaxanthin	Guinea pig, zebrafish	Gu et al. (2020)
	PEG	Intradermal	mPEG-PCL nanoparticles containing artemisinin	Guinea pig	Li et al. (2020)
Inorganic	Iron oxide	Pore induction in RWM	Delivery of iron oxide nanoparticles using micro-shotgun induced pores in the RWM	Guinea pig	Liang et al. (2020)
	Dendrimer	Intratympanic	Polyamidoamine dendrimers containing Atoh1 plasmid gene therapy	Rat	Wu et al. (2013)
	Gold	Pore induction in RWM	Delivery of chitosan-coated gold nanoparticles using ultrasound microbubble-induced pores in the RWM	Mouse	Lin et al. (2021)
	Silica	Intracochlear, RWM administration	Mesoporous silica supraparticles containing neurotrophins, brain-derived neurotrophic factor	Guinea pig	Wang et al. (2014b), Wise et al. (2016), Gunewardene et al. (2022)

## 6 Novel drug delivery systems

### 6.1 Hydrogels

Hydrogel delivery systems allow for the controlled release of a variety of therapeutic compounds. They can be manipulated into virtually any shape or size to facilitate controlled drug release and delivery to various locations in the body (Li and Mooney, 2016; Rathnam et al., 2019). For hearing-based therapies, the most common form tested preclinically is direct application of a liquid *in situ* gelling hydrogel. The hydrogel is administered to the TM or round window niche via intratympanic injection, which solidifies upon contact with the RWM (Rathnam et al., 2019). This allows for the continual and controlled release of drugs which diffuse across the RWM to the inner ear, without a need for highly invasive surgery (Paulson et al., 2008; Hütten et al., 2014; Lajud et al., 2015; Chen et al., 2022d).

Most hydrogel systems are advantageous due to their biocompatibility. For example, gelatin methacryloyl (GelMA) is highly biocompatible and has been extensively studied in the laboratory as a substrate for three-dimensional mammalian cell culture due to its inert nature, tunability, and higher batch-to-batch reproducibility over other commonly used substrates (Zhu et al., 2019). In an interesting recent approach for clinical application, GelMA hydrospheres were conjugated with polydopamine (PDA) for cell adhesion. The delivery of ebselen via these GelMA hydrospheres recovered hearing loss at some frequencies in a mouse model of NIHL (Chen et al., 2022d). Another biocompatible hydrogel composed of modified polyethylene glycol (PEG) was used to deliver a hydrophilic form of dexamethasone to a guinea pig cochlear trauma model, resulting in sustained drug delivery over several weeks, causing the recovery of hearing loss and reduced fibrosis (Hütten et al., 2014).

Additional classes of hydrogel include poloxamers, with a recent approach using the commercially available Poloxamer 407 to deliver a small molecule inhibitor of the apoptosome, LPT99. This approach was shown to preserve cell viability and auditory function in cisplatin-treated rats (Murillo-Cuesta et al., 2021). Finally, polylactic-co-glycolic acid (PLGA) hydrogels have shown promise due to their biodegradable nature and have potential to deliver a wide variety of drugs, including large molecules such as proteins (Dai et al., 2018; Kim et al., 2021). However high doses of a PLGA-PEG-PLGA copolymer were observed to affect hearing in guinea pigs (Feng et al., 2014).

Otiprio^®^ is an FDA-approved ciprofloxacin solution suspended in a thermosensitive Poloxamer 407 gel and has been indicated primarily for the treatment of pediatric otitis externa and OME associated with tympanostomy tube placement (Edmunds, 2017). Otiprio^®^ is thought to be superior to conventional ciprofloxacin eardrops (which usually requires three drops thrice a day) as it has been shown to reduce the occurrence of OM after a single intratympanic injection (Renukananda et al., 2014; Dohar et al., 2018). While Otriprio^®^ is not currently approved for the treatment of CSOM, it is a promising “off-label” treatment due to the similarity of the two disease subtypes.

There are several hydrogel-based therapies for SNHL that are currently undergoing or have completed clinical trials. One of the earliest was developed by Otonomy termed “OTO-104”, a poloxamer gel injected intratympanically to deliver dexamethasone across the RWM to the inner ear (Piu et al., 2011). This therapy underwent Phase 2 and 3 clinical trials for vertigo in cisplatin-induced hearing loss and Meniere’s disease respectively, however both were terminated once no significant difference was observed compared to placebo for the former trial. This has been postulated to be due to accelerated clearance of the drug, as dexamethasone can readily cross the BLB and may be eliminated prior to affecting the more apical regions of the cochlea (Salt and Plontke, 2018). More recent developments include DB-020 and FX-322 from Decibel Therapeutics and Frequency Therapeutics respectively. DB-020 is a hyaluronic acid-based gel containing sodium thiosulfate for the inactivation of cisplatin, an ototoxic chemotherapy. Phase 1 clinical trials showed DB-020 was well tolerated and that 13 of 17 patients treated displayed partial or complete otoprotection (Viglietta et al., 2020). The progression and further studies of DB-020 will be useful to determine if the hydrogel confers additional otoprotection or higher tolerability in patients than the intravenously injected formulation recently approved by the U.S. Food and Drug Administration (Brock et al., 2018). FX-322 is a poloxamer-based gel containing the glycogen synthase kinase inhibitor, CHIR99021 and the histone deacetylase inhibitor, valproic acid. Both have been shown to promote the differentiation of stem cells into an otic lineage (Koehler et al., 2017). FX-322 showed a significant improvement in patients with chronic SNHL compared to the placebo in Phase 1b clinical trials (McLean et al., 2021). Unfortunately, Frequency Therapeutics reported that FX-322 showed no statistically significant difference from placebo at day 90 of a Phase 2b trial in February 2023 and development of the drug was halted.

Hydrogels have great potential to facilitate controlled delivery of a wide variety of drugs, including nanoparticles which will be discussed in the next section, and are an appealing avenue based on their ever-expanding tunability and composition. Despite these advantages, some precluding factors remain in their progression to more prevalent clinical usage. Namely, sterilization and storage could be challenging given their hydrated nature (Li and Mooney, 2016). Nevertheless, the degree of controlled release afforded by hydrogel drug delivery remains a promising factor in their applicability to treat hearing loss.

### 6.2 Nanoparticles

Due to their small size and theoretically endless possibilities for customization, nanoparticles have become an attractive option for drug delivery (Table 1). The ability to customize nanoparticles is advantageous as size, charge, lipid solubility and membrane thickness strictly govern molecular passage across the RWM and these properties cannot be readily modified in most drugs (Goycoolea and Lundman, 1997; Valente et al., 2017; Xu et al., 2021a; Xu et al., 2021b). Nanoparticles of sizes between 10 and 640 nm have been demonstrated to diffuse across the RWM, however most studies concur that sizes below 200 nm display the greatest permeation (Valente et al., 2017; Xu et al., 2021a; Chester et al., 2021).

Regarding charge, positively-charged nanoparticles display higher diffusion and greater distribution in the apical region of the cochlea, which is likely due to the ability of more positively charged nanoparticles to cross the lipid membranes and plasma membranes into cells (Liu et al., 2013; Yang et al., 2018a). A study using nanoparticles coated with a positively-charged arginine 8 peptide shell exploited this to deliver connexin 26 siRNA and plasmids containing green fluorescent protein or brain-derived neurotrophic factor (BDNF), along with dexamethasone, which induced nuclear pore opening (Yoon et al., 2016). Another study demonstrated greater distribution of cationic lipid nanoparticles in an *in vitro* RWM model, over neutrally charged or anionic nanoparticles of the same type. This nanoparticle also mitigated against an induced inflammatory reaction *in vivo* (Yang et al., 2018b).

Moreover, like hydrogels, nanoparticles can provide a means of controlled drug release in the middle and inner ear. This could decrease the need for surgical intervention for routine application of therapeutics and decrease potential systemic toxicity. Thus, nanotechnology has become of great recent interest in drug delivery to the inner ear.

#### 6.2.1 Lipid nanoparticles

Lipid nanoparticles are generally composed of amphipathic lipids, such as phospholipids. Phospholipids confer biocompatibility by allowing incorporation of the lipid nanoparticle into the cell membranes via endocytosis, micropinocytosis or degradation (Zou et al., 2017; Xu et al., 2021b; Chester et al., 2021; Lu et al., 2021). Furthermore, except for micelles, most lipid nanoparticle types can simultaneously encapsulate hydrophilic and lipophilic drugs in their aqueous region and lipid tail regions, respectively.

One of the original types of lipid nanoparticle are liposomes, which comprise an amphipathic lipid bilayer encapsulating a therapeutic payload. Liposomes were one of the first lipid nanoparticles to be clinically tested, however this class of nanoparticle has only been tested in the inner ear relatively recently in preclinical models. In a recent example by Zou et al. (2017), the biocompatibility of a liposome nanoparticle in rats was assessed through gadolinium MRI imaging which revealed no structural change to the inner ear and histology, which showed no cell death or inflammation, aside from a slight increase in the secretion of the extracellular matrix protein, hyaluronan. Another pre-clinical application of liposomes, delivered CRISPR Cas9-guide RNA complexes to target the pathogenic allele in the *Bth* mouse model of DFNA36 autosomal dominant deafness, using the commercially available Lipofectamine 2000 (Gao et al., 2018a). Lipofectamine 2000 is a liposome comprised of cationic lipids, but is more commonly used as a transfection reagent in laboratory settings (Dalby et al., 2004). Moreover it is slightly cytotoxic, so ideally a more biocompatible vector would be suitable for CRISPR-Cas or gene delivery (György et al., 2019). Nevertheless, this demonstrates the applicability of lipid nanoparticles for the delivery of a wide variety of therapeutics.

**FIGURE 2:**
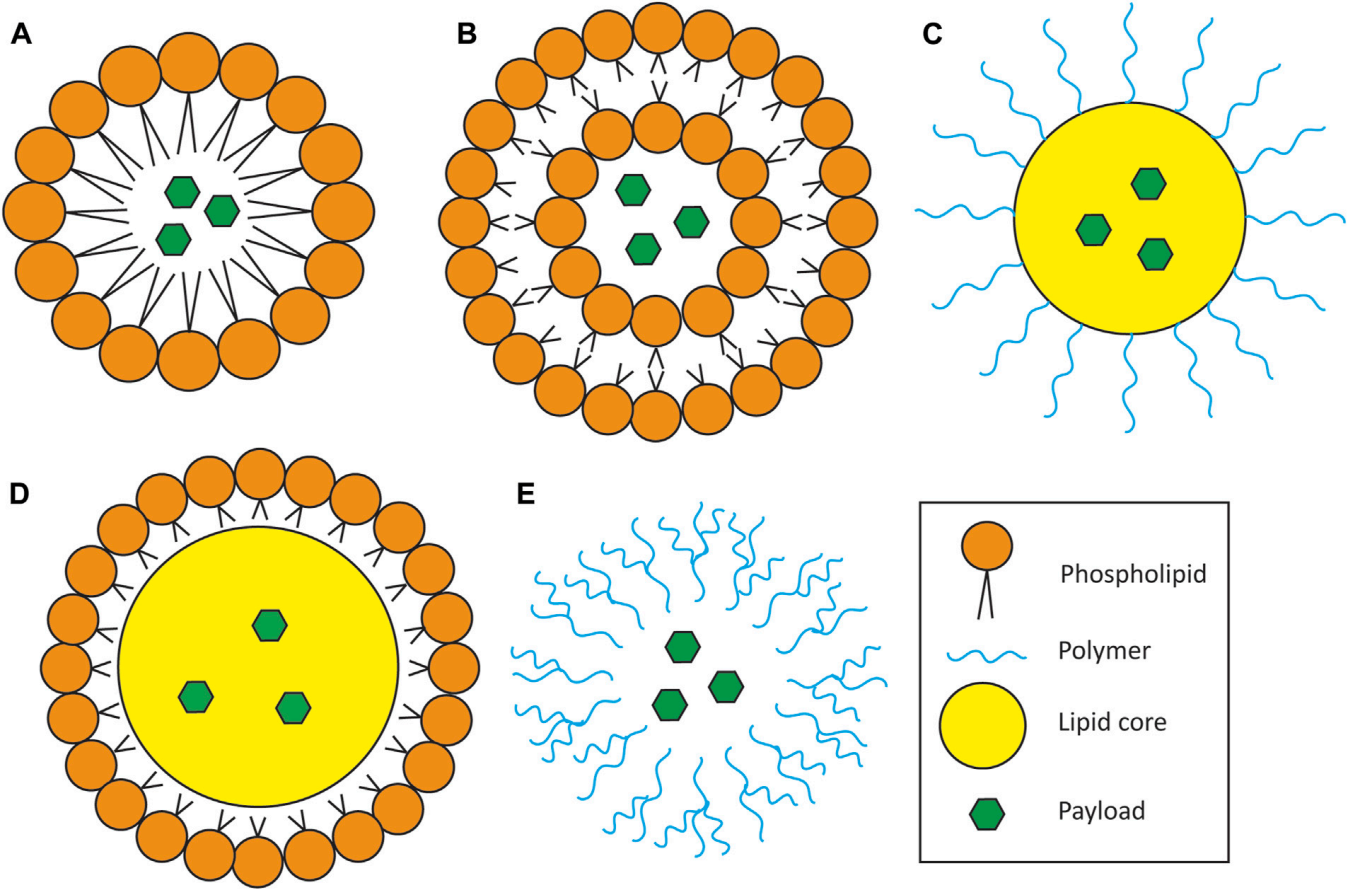
Schematic of nanoparticle types for drug delivery. **(A)** Micelles. Micelles consist of a single layer of amphipathic lipids surrounding a lipophilic payload. **(B)** Liposomes. Liposomes consist of an amphipathic lipid bilayer encapsulating either or both a hydrophilic payload in the core and/or a lipophilic payload inside the lipid bilayer (latter not depicted in cartoon). **(C,D)** Lipid core nanoparticle. Characterized by an outer shell of polymers **(C)** or other lipids **(D)** surrounding a lipid droplet core containing the therapeutic payload. **(E).** Polymeric nanoparticle. Made entirely of polymers and can carry both hydrophilic and lipophilic payloads depending on the polymer used. This particular depiction represents dendrimers, which consist of long, branched chains of polymers encapsulating the therapeutic payload.

Other types of lipid nanoparticles include nanoemulsions, lipid core nanoparticles which have a lipid core enclosed by a shell of biocompatible polymers for stability, and solid-lipid nanoparticles (Figure 2)**.** Solid-lipid nanoparticles (SLN) are composed of solid lipids, emulsifiers and water, and are of the most recently investigated for use in hearing loss. SLNs offer many advantages over other nanoparticle types. These include controlled drug release, stability at room temperature, higher payload, low toxicity and the ability to be functionalized and sterilized (Mehnert and Mäder, 2012). Recently, steric acid-based SLNs containing dexamethasone or hydrocortisone were shown to internalize in HEI-OC1 cells without compromising cell viability and additionally were otoprotective against cisplatin treatment (Cervantes et al., 2019). Other preclinical reports of SLN-mediated cochlear protection include the delivery of the antioxidant edaravone (Gao et al., 2015) and the antipsychotic clozapine (Wang et al., 2020), where both were shown to protect against NIHL in guinea pigs.

#### 6.2.2 Polymeric and inorganic nanoparticles

There is much larger variety in polymeric nanoparticles, which are composed of long and branched or linear networks. These can be manufactured structurally similar to lipid nanoparticles, allowing for the encapsulation of both hydrophilic and lipophilic payloads. Polymeric nanoparticles have also been more widely investigated in inner ear therapy, offering better reproducibility and easier surface functionalization than most lipid nanoparticles. However, the large molecular weight of their polymer components carries higher risks in immunogenicity and clearance, and may decrease the amount of payload that can be delivered (Xu et al., 2021b; Lu et al., 2021).

Many hydrogel components can also constitute polymeric nanoparticles and in some instances, the terms “nanoparticle” and “nanogel” are interchangeable (Rathnam et al., 2019). For example, nanoparticles comprised of PLGA were shown to have increased RWM diffusion and cochlear penetration when polymerized with Poloxamer 407, PEG or chitosan, all of which are well-characterized hydrogel components (Cai et al., 2017). Moreover, PEG-based nanoparticles, loaded with artemisinin as an antioxidant, were shown to provide some protection against gentamicin-induced ototoxicity (Li et al., 2020). Another recent approach involved the polymerization of PEG and the commercially available DMPC phospholipid. The resulting lipid polymer nanoparticle was used to deliver the antioxidant astaxanthin, which alleviated the toxic effects of cisplatin on HEI-OC1 cells, as well as guinea pig and zebrafish outer hair cells (Gu et al., 2020). Finally, another PEG nanoparticle-based strategy used a bifunctionalized nanoparticle incorporating poly (propylene sulfide)_120_, a scavenger of reactive oxygen species, and an outer hair cell targeting peptide to deliver the Chinese herbal medicine berberine into a guinea pig model of NIHL (Zhao et al., 2021).

Dendrimers have been of recent interest for delivery of non-viral gene therapy in many areas, including SNHL. Dendrimers are nanoparticles characterized by long, branched polymer chains and are readily customizable in terms of their molecular architecture, which confers the ability to carry a wider variety of drugs and allows surface functionalization, for example for conjugating permeation or targeting moieties (Kheiriabad et al., 2021). One study demonstrated greater gene transfection efficiency of a polyamidoamine dendrimer over the commercially available transfection reagent, Lipofectamine 2000, a lipid nanoparticle (Gao et al., 2018b). Another polyamindoamine-based dendrimer was used to deliver an *ATOH1*-containing plasmid to the rat inner ear. The dendrimer was shown to permeate across the RWM and transfer the plasmid to hair cells (Wu et al., 2013). Moreover, dendrimers have shown promise in delivering CRISPR-Cas systems in other applications, demonstrating their future potential in gene therapy for the inner ear (Kretzmann et al., 2019).

Nanoparticles have additionally shown promise for the treatment of chronic middle ear disorders. Two recent studies using gold nanoclusters has shown specific targeting of antibiotic-resistant persister cells. The first used the nanoclusters as an adjuvant therapy with ofloxacin. The combination therapy sterilized *in vitro* biofilms and significantly reduced *P. aeruginosa* numbers in a mouse model of CSOM without inducing ofloxacin resistance (Cao et al., 2022). The second study functionalized the gold nanoclusters with adenosine triphosphate and showed significant lethal effect on metabolically inactive bacteria (Bekale et al., 2023). Both approaches showed the nanoclusters induced cell death through disruption of outer membrane integrity by various mechanisms and were well tolerated *in vivo*.

One interesting approach for cholesteatoma used indocyanine green nanocapsules to target cholesteatoma-derived keratinocytes. This approach is designed as an adjuvant therapy to eradicate residual cells left behind after surgery. The nanocapsules were coated with an antibody against epidermal growth factor receptor (EGFR) to selectively target the keratinocytes. *In vitro* results showed selective targeting and killing of keratinocytes, but not mucosal cells after activation of the nanocapsules with infrared light (Gluth et al., 2015).

#### 6.2.3 Nanoparticle delivery to the inner ear

While nanoparticles address controlled release of drugs in the inner ear, ultimately they must themselves be delivered to the inner ear. We have addressed a subset of nanoparticles that have demonstrated ability to cross the RWM, however there are many other classes of nanoparticle that have shown therapeutic potential for the treatment of hearing loss. These are usually larger nanoparticles with larger payloads or those that can be externally directed once implanted.

Nanoparticles can be engineered with inherently magnetic properties. Superparamagnetic iron oxide nanoparticles are already used in the clinic, with many applications in imaging, such as MRI (Dadfar et al., 2020). While it may be difficult to precisely deliver magnetic nanoparticles to the inner ear via the circulation, magnetic approaches have been proposed to deliver drugs to the inner ear locally. This is an advantageous approach as the drugs would be actively delivered into the inner ear, rather than through passive diffusion. One study demonstrated this by showing penetration of superparamagnetic nanoparticles into the scala tympani and apex of the cochlea, when magnetically directed after application onto the RWM in a hyaluronic acid gel (Leterme et al., 2019). Another approach used magnetic nanoparticles to deliver prednisolone into the cochlea of cisplatin-treated mice, which showed significant otoprotection compared to control or intratympanic injection (Ramaswamy et al., 2017).

As previously discussed, limitations on drugs and nanoparticles administered on the RWM include size, charge, lipophilicity and membrane thickness. However, as nanoparticles can allow for controlled release of drugs once injected, invasive surgeries such as intracochlear injection, would not be repeatedly required once the nanoparticle is delivered. Intracochlear injection would additionally allow the delivery of larger drugs, including large nanoparticles, microparticles and therapeutic peptides.

An example of intracochlear injection for large therapeutic delivery involves the administration of mesoporous silica supraparticles (MS-SP) containing neurotrophins. These MS-SPs are comprised of MS nanoparticles which self-assemble into larger SPs capable of delivering larger payloads, with pore sizes of 15–30 nm for neurotrophins (Wang et al., 2014b). Intracochlear injections of MS-SPs containing BDNF (Wise et al., 2016) or neurotrophin-3 (Gunewardene et al., 2022) were shown to recover damage to auditory nerves and the organ of Corti in deafened guinea pigs, while eliciting minimal inflammatory response. Furthermore, the use of MS-SPs achieved controlled release of the neurotrophins up to a month post-injection, though the authors noted some loss of hearing at higher frequencies caused by intracochlear injection when compared with RWM administration (Gunewardene et al., 2022). These studies highlight the advantages and risks associated with intracochlear injection.

The delivery of nanoparticles can additionally be enhanced through hydrogel-mediated release. As discussed previously, drugs contained in hydrogels that are implanted on the RWM reach the inner ear by passive diffusion through the membrane. Nano-encapsulation could provide an extra layer of controlled release and ensure these drugs are not compromised after RWM diffusion. For example, PEG-based nanoparticles containing a JNK inhibitor were delivered using a chitosan/glycero-2-phosphate (CGP) hydrogel implanted through intratympanic injection. These nanoparticles were functionalized by adding a peptide targeting prestin, a protein exclusively expressed by outer hair cells, and was shown to provide protection against NIHL (Kayyali et al., 2018). A previous study by this group showed the ability to abrogate drug delivery of the hydrogel upon treatment with chitosanase, which digests the CGP hydrogel (Lajud et al., 2013).

Some creative methods of introducing transient pores in the RWM have been designed for the delivery of nanoparticles into the inner ear. A “micro-shotgun” was developed to transport iron oxide and chitosan-based nanoparticles through the RWM. Briefly, this was constructed by loading nanoparticles and sodium carbonate/citric acid “fuel” into a microtube which was guided using a magnetic field and iron oxide nanoparticles in the microtube. The addition of water to the loaded tube causes a reaction generating a large amount of carbon dioxide, which launches the nanoparticle. Pores in the guinea pig RWM created by the launch closed 24 h after with no injury to the stria vascularis, organ of Corti or spiral ganglion neurons observed (Liang et al., 2020). Another vastly different approach used ultrasound microbubbles to deliver chitosan-coated gold nanoparticles. These microbubbles caused a transient disruption of tight junctions in the RWM upon light sonication, which facilitated the entry of chitosan-coated gold nanoparticles through the RWM (Lin et al., 2021).

Nanoparticle-based treatment is an emerging area with a multitude of new preclinical studies being reported. Nanoparticles address many challenges in therapeutic delivery to the inner ear, with some formulations having the ability to traverse the RWM and effectively distribute along the cochlea. However, key areas need be addressed, including drug release kinetics and clearance, with many studies cited in this review focusing on penetration into the inner ear without the nanoparticle necessarily carrying a therapeutic payload. There are additionally some cell-protective mechanisms by which nanoparticles can be prevented from reaching their therapeutic target, most notably endosomal escape (Smith et al., 2019). Furthermore, challenges in large scale production and sterilization remain depending on the type of nanoparticle. Nevertheless, advances in nanoparticle technology have created the possibility of the transport of drugs that cannot penetrate the RWM, such as proteins and nucleic acid-based therapies for hearing loss. The field continues to rapidly expand, increasing our understanding of how to deliver nanoparticles and new forms of drugs into the inner ear.

### 6.3 Homing peptides

Homing peptides are widely used to deliver therapeutic payloads to tumors, diseased blood vessels and to treat neurological disorders. These peptides use “molecular zip codes” or cell/extracellular matrix recognition motifs to selectively identify cell surface markers and extracellular matrix, which permit accumulation of the peptide in not only specific tissues, but specific cells (Ruoslahti, 2022). Homing peptides additionally can be conjugated to a therapeutic cargo to guide the payload to the site of pathology (Mann et al., 2016; Yeow et al., 2019). They are commonly generated from *ex vivo*/*in vivo* phage display screening, where bacteriophages are incubated with cells from target tissue, generating a library of potential homing peptides on the phage surface. These peptides are generally small, usually up to 20–30 amino acids in length, which allows greater permeation across membranes than larger targeting moieties such as antibodies.

Recently, Allen Ryan’s group at the University of California, San Diego used this technique to identify homing peptides that could cross the TM. The *ex vivo* study used TM from rats with induced OM as the substrate for peptide identification, which produced candidate peptides which were able to transport the phage across the TM *in vivo* (Kurabi et al., 2018a). A follow up *ex vivo* study showed the homing peptides were able to cross human TM both as isolated peptides and connected to phage at the same rate (Kurabi et al., 2018b). The mechanism of transport was eventually implicated to be via transcytosis (Kurabi et al., 2022). Based on the relatively large size of bacteriophages (900 nm-1 µm in length), these would make homing peptides a highly promising approach for the delivery of larger drugs and nanoparticles to the middle and potentially inner ear.

## 7 Microdevice-mediated delivery

The aforementioned intratympanic approach generally relies on passive diffusion for the drug to cross the RWM and reach its target. This may not be sufficient to achieve an adequate therapeutic concentration for some drugs and approaches designed to allow reloading of drug to a surgically-implanted device are under development. These devices would be relatively easy to access or “reload” from the outside and be directly connected to a compartment within the middle or inner ear. One of the earliest examples of such a device in clinical use is the Silverstein MicroWick, which was developed by Herbert Silverstein and colleagues. The MicroWick is a polyvinyl acetate catheter threaded through a ventilation tube which is surgically-installed in the TM. The catheter contacts the RWM and drugs such as gentamicin and dexamethasone can be self-administered as eardrops up to three times daily for treatment of Meniere’s disease and sudden deafness respectively (Silverstein et al., 2004). More complex solutions involving continual delivery have since been envisioned to cater for a greater variety of therapeutics, as the MicroWick must be removed after 4 weeks and may not be able to deliver larger or more sensitive drugs, such as therapeutic peptides or gene therapies.

### 7.1 Micropumps, direct perfusion and reciprocating systems

Miniaturized perfusion systems have been trialed in animal models as a possible delivery device for drugs. Directed intracochlear delivery overcomes many limitations of intratympanic RWM administration. Larger and more unstable drugs such as RNA therapeutics or proteins can be continuously delivered and multiple doses can be accounted for with the continual delivery. However, introducing more fluid must be precise and delivered in small volumes to not risk increasing intracochlear pressure and damaging sensory tissue. Moreover, the design of the preclinical systems thus far suggests a direct translation of a wearable or implanted pump for human use. Such systems would necessitate the highest practical degree of miniaturization to avoid inconveniencing the patient and reduce any possible stigma if the device is visible, as those with hearing aids may experience (Wallhagen, 2009).

Jeffrey Borenstein’s group has been extensively involved in the design of micropumps for inner ear delivery. In 2015, they reported the design of a head-mounted micropump (using guinea pigs for demonstration), with infuse-withdraw capabilities. This functioned through the infusion-withdrawal of perilymph so as to not disturb intracochlear pressure and was used to deliver the glutamate receptor antagonist DNQX (Tandon et al., 2015). The group also created a device with a reciprocating pump and drug reservoir to ensure no net volume change in the cochlea (Kim et al., 2014). Another group was able to recently create an implantable micropump for mice (Forouzandeh et al., 2019).

### 7.2 Cochlear prosthesis-mediated delivery and mitigation of cochlear fibrosis

Cochleostomy is a highly invasive process, which itself may risk affecting hearing. This procedure is usually employed to install cochlear implants and there is growing *in vivo* evidence in animal models and humans of a fibro-inflammatory response to implants (Wilk et al., 2016; Foggia et al., 2019). The resulting fibrosis interferes with the implant electrode array, and is thought to contribute to impedance, which hampers the effectiveness of the cochlear implant over time. To counteract the proliferation of fibrotic tissue, anti-inflammatory drugs, commonly dexamethasone, incorporated into the implant electrode array have shown effectiveness at preventing fibrosis and additionally trauma-induced hair cell and neural damage (Chang et al., 2009; Eastwood et al., 2010; Bas et al., 2016; Wilk et al., 2016; Plontke et al., 2017; Qnouch et al., 2021). Coating the surface of the implant with less adsorbing and immunogenic biomaterials has also shown benefit in inhibiting the inflammatory response (Chen et al., 2022a).

As discussed in previous sections however, certain drugs, especially dexamethasone and other glucocorticoids, risk being cleared prior to achieving sufficient bioavailability (Salt and Plontke, 2018). A study by Liebau et al. (2020) demonstrated this by implanting silicone dummy rods with different concentrations of dexamethasone, showing a dose-dependent relationship between drug loading amount and perilymph concentration over 7 weeks in guinea pigs. To ensure continual release of drugs, some compelling controlled release strategies have been developed. One study used Pluronic-coated gold nanoparticles to deliver dexamethasone in the round window niche after cochleostomy and demonstrated modest improvement in ABR threshold at 8 kHz, but no difference at other ranges (Blebea et al., 2022). Another study from Chen et al. (2022b) presented an interesting strategy using mesoporous silica nanoparticles containing siRNA against the pro-fibrotic cytokine, TGFβ, which were adsorbed onto the surface of the electrode array. *In vivo* results from animal studies from this group are highly anticipated.

## 8 Conclusion

We have reviewed existing clinical problems and recent innovations in drug delivery to the middle and inner ear. While drug delivery to the middle and inner ear remains a significant clinical problem, great advances have been made recently. As the anatomical barriers to the middle and inner ears preclude straightforward local delivery, it will likely require a combinatorial approach of the techniques mentioned to ensure effectiveness of these novel technologies. For example, intratympanic hydrogel administration of many drugs, including corticosteroids by themselves, is insufficient due to rapid clearance from the cochlea (Salt and Plontke, 2018). However, a dual-delivery system that utilizes a hydrogel and nanoencapsulation of drugs could counteract the rapid clearance rates in the middle and inner ear. Also targeting moieties, such as homing peptides, could ensure the administered drug reaches its cellular or molecular target once administered in the relatively static cochlear fluids (Kayyali et al., 2018; Ruoslahti, 2022).

An additional challenge for the field is how the development of drug delivery systems keep up with the development of novel treatment types. Many gene therapies for example, are relatively unstable molecules and must be kept in a medium whereby they remain viable once they reach the inner ear. Conventional gene therapies, for example the delivery of whole genes, may present a problem based on molecular size. This is common and reasonably expected for those replacing the large multidomain proteins of the cochlear stereocilia bundles, and already challenges the limited capacity of the more conventional gene delivery system of adeno-associated viruses (French et al., 2020). This presents an opportunity for non-viral gene delivery systems such as nanoparticles, however nanoparticles themselves are also constrained by their own size when considering intratympanic RWM delivery and passage (Mehnert and Mäder, 2012). Furthermore, technical considerations such as sterility and dosage consistency between nanocapsules of these more sensitive therapies need to be considered.

The emergence of biomaterial-based delivery systems, particularly hydrogels, in mid-late stage clinical trials demonstrates the potential for these novel systems to become mainstream options for local middle and inner ear drug delivery. Future studies should continue to address drug release and pharmacokinetics of encapsulated drugs in animal models of hearing loss in terms of tissue penetration and clearance. Moreover, the development of reliable *in vitro* RWM models would additionally be useful for large scale assessment of drug penetration. These types of studies would ensure the maintenance of drug safety and efficacy as other promising delivery systems are developed.
